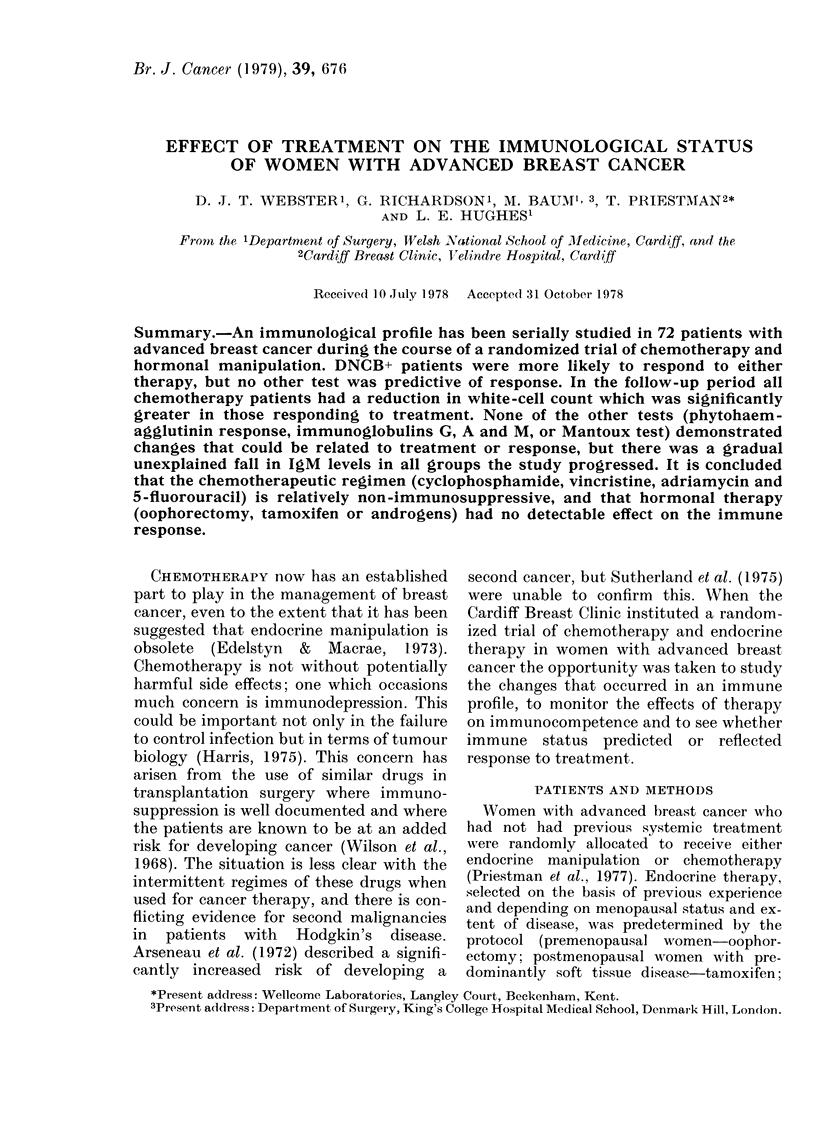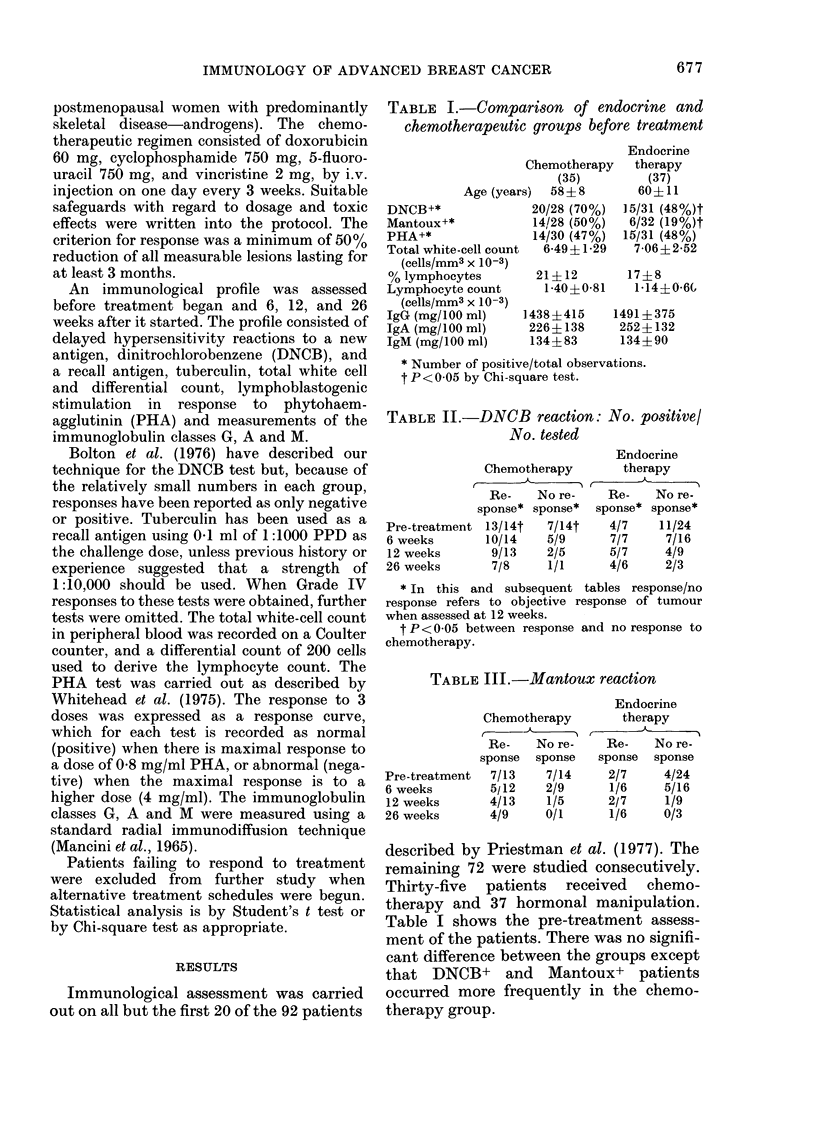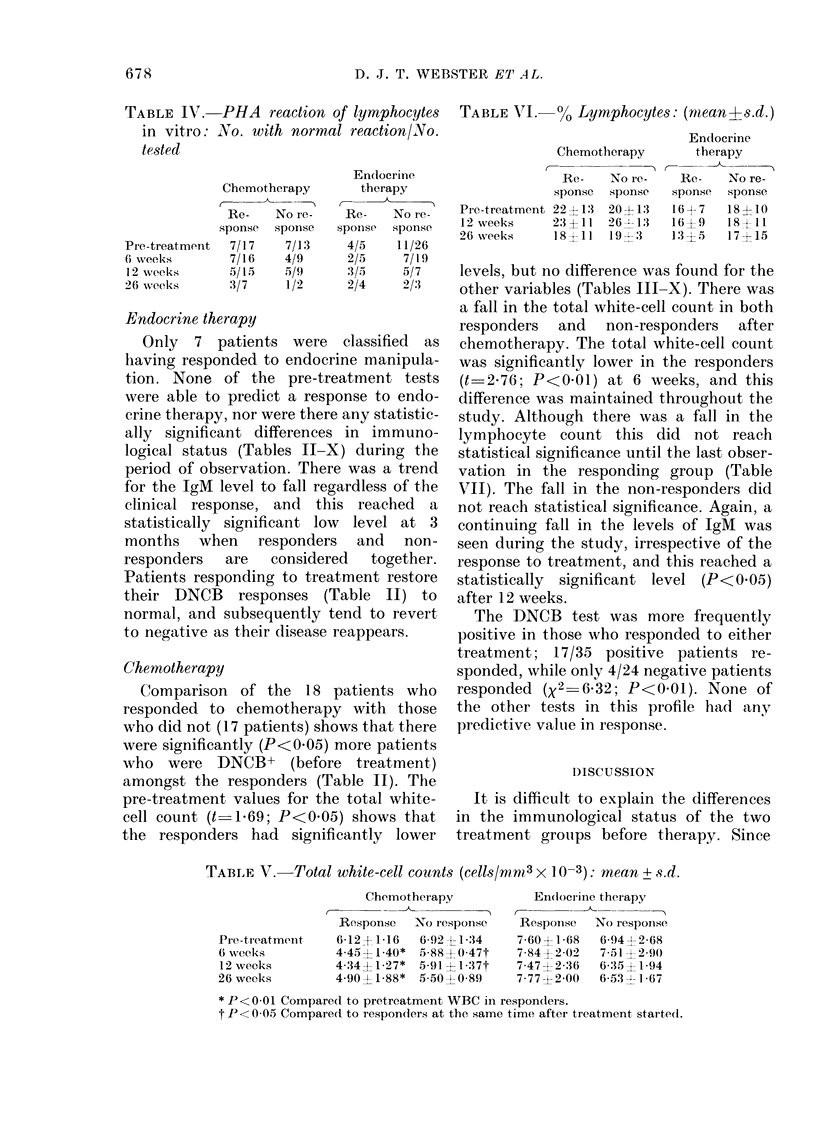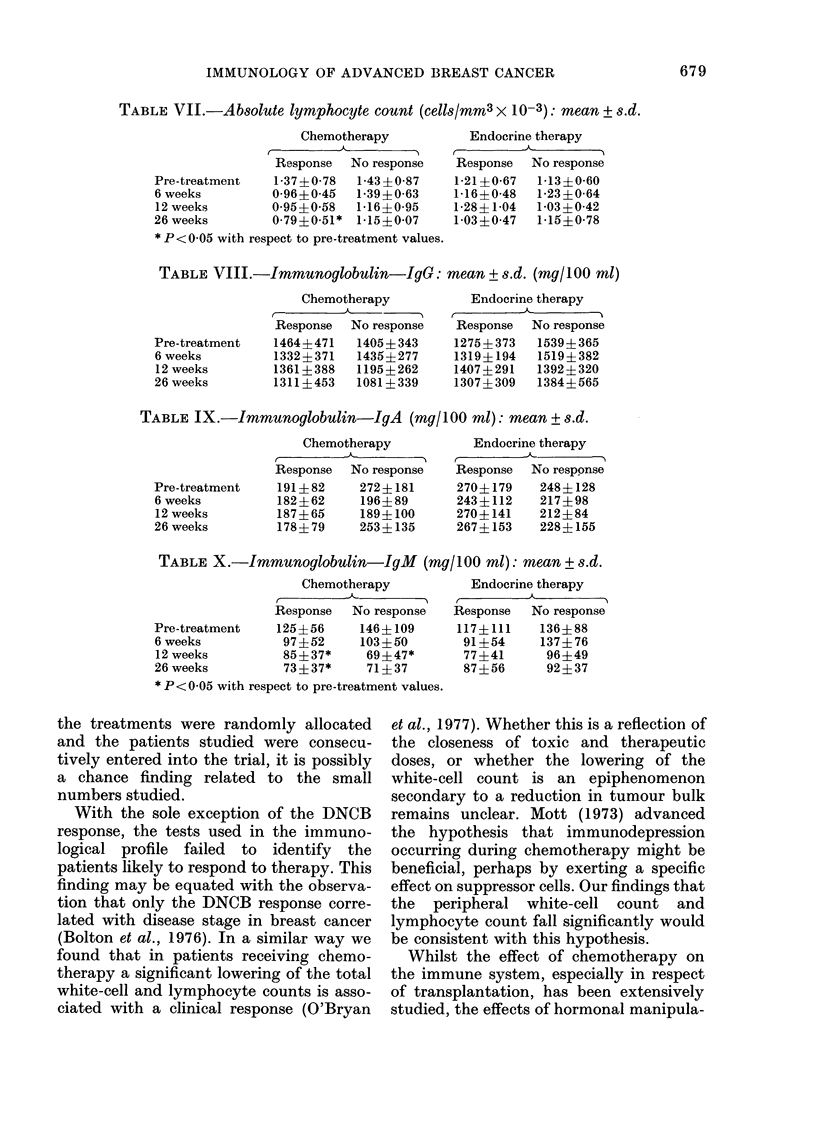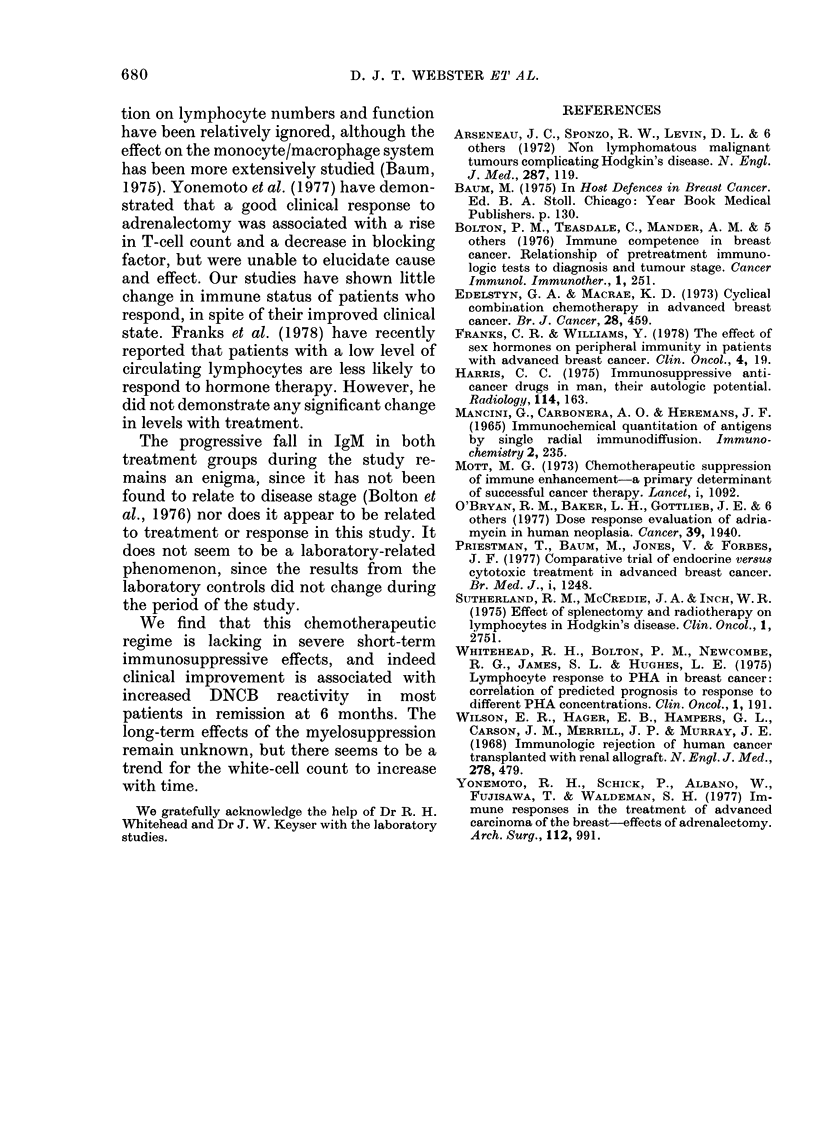# Effect of treatment on the immunological status of women with advanced breast cancer.

**DOI:** 10.1038/bjc.1979.119

**Published:** 1979-06

**Authors:** D. J. Webster, G. Richardson, M. Baum, T. Priestman, L. E. Hughes

## Abstract

An immunological profile has been serially studied in 72 patients with advanced breast cancer during the course of a randomized trial of chemotherapy and hormonal manipulation. DNCB+ patients were more likely to respond to either therapy, but no other test was predictive of response. In the follow-up period all chemotherapy patients had a reduction in white-cell count which was significantly greater in those responding to treatment. None of the other tests (phytohaemagglutinin response, immunoglobulins G, A and M, or Mantoux test) demonstrated changes that could be related to treatment or response, but there was a gradual unexplained fall in IgM levels in all groups the study progressed. It is concluded that the chemotherapeutic regimen (cyclophosphamide, vincristine, adriamycin and 5-fluorouracil) is relatively non-immunosuppressive, and that hormonal therapy (oophorectomy, tamoxifen or androgens) had no detectable effect on the immune response.


*Br. J. Cancer* (1979), **39**, 676

### EFFECT OF TREATMENT ON THE IMMUNOLOGICAL STATUS OF WOMEN WITH ADVANCED BREAST CANCER

D. J. T. WEBSTER[1], G. RICHARDSON[1], M. BAUM[1, 3], T. PRIESTMAN[2]* AND L. E. HUGHES[1]

*From the [1]Department of Surgery, Welsh National School of Medicine, Cardiff, and the [2]Cardiff Breast Clinic, Velindre Hospital, Cardiff*

Received 10 July 1978 Accepted 31 October 1978

**Summary.—An immunological profile has been serially studied in 72 patients with advanced breast cancer during the course of a randomized trial of chemotherapy and hormonal manipulation. DNCB+ patients were more likely to respond to either therapy, but no other test was predictive of response. In the follow-up period all chemotherapy patients had a reduction in white-cell count which was significantly greater in those responding to treatment. None of the other tests (phytohaemagglutinin response, immunoglobulins G, A and M, or Mantoux test) demonstrated changes that could be related to treatment or response, but there was a gradual unexplained fall in IgM levels in all groups the study progressed. It is concluded that the chemotherapeutic regimen (cyclophosphamide, vincristine, adriamycin and 5-fluorouracil) is relatively non-immunosuppressive, and that hormonal therapy (oophorectomy, tamoxifen or androgens) had no detectable effect on the immune response.**

CHEMOTHERAPY now has an established part to play in the management of breast cancer, even to the extent that it has been suggested that endocrine manipulation is obsolete (Edelstyn & Macrae, 1973). Chemotherapy is not without potentially harmful side effects; one which occasions much concern is immunodepression. This could be important not only in the failure to control infection but in terms of tumour biology (Harris, 1975). This concern has arisen from the use of similar drugs in transplantation surgery where immunosuppression is well documented and where the patients are known to be at an added risk for developing cancer (Wilson *et al.*, 1968). The situation is less clear with the intermittent regimes of these drugs when used for cancer therapy, and there is conflicting evidence for second malignancies in patients with Hodgkin's disease. Arseneau *et al.* (1972) described a significantly increased risk of developing a second cancer, but Sutherland *et al.* (1975) were unable to confirm this. When the Cardiff Breast Clinic instituted a randomized trial of chemotherapy and endocrine therapy in women with advanced breast cancer the opportunity was taken to study the changes that occurred in an immune profile, to monitor the effects of therapy on immunocompetence and to see whether immune status predicted or reflected response to treatment.

### PATIENTS AND METHODS

Women with advanced breast cancer who had not had previous systemic treatment were randomly allocated to receive either endocrine manipulation or chemotherapy (Priestman *et al.*, 1977). Endocrine therapy, selected on the basis of previous experience and depending on menopausal status and extent of disease, was predetermined by the protocol (premenopausal women—oophorectomy; postmenopausal women with predominantly soft tissue disease—tamoxifen;

*Present address: Wellcome Laboratories, Langley Court, Beckenham, Kent.
[3]Present address: Department of Surgery, King's College Hospital Medical School, Denmark Hill, London.

postmenopausal women with predominantly skeletal disease—androgens). The chemotherapeutic regimen consisted of doxorubicin 60 mg, cyclophosphamide 750 mg, 5-fluorouracil 750 mg, and vincristine 2 mg, by i.v. injection on one day every 3 weeks. Suitable safeguards with regard to dosage and toxic effects were written into the protocol. The criterion for response was a minimum of 50% reduction of all measurable lesions lasting for at least 3 months.

An immunological profile was assessed before treatment began and 6, 12, and 26 weeks after it started. The profile consisted of delayed hypersensitivity reactions to a new antigen, dinitrochlorobenzene (DNCB), and a recall antigen, tuberculin, total white cell and differential count, lymphoblastogenic stimulation in response to phytohaemagglutinin (PHA) and measurements of the immunoglobulin classes G, A and M.

Bolton *et al.* (1976) have described our technique for the DNCB test but, because of the relatively small numbers in each group, responses have been reported as only negative or positive. Tuberculin has been used as a recall antigen using 0·1 ml of 1:1000 PPD as the challenge dose, unless previous history or experience suggested that a strength of 1:10,000 should be used. When Grade IV responses to these tests were obtained, further tests were omitted. The total white-cell count in peripheral blood was recorded on a Coulter counter, and a differential count of 200 cells used to derive the lymphocyte count. The PHA test was carried out as described by Whitehead *et al.* (1975). The response to 3 doses was expressed as a response curve, which for each test is recorded as normal (positive) when there is maximal response to a dose of 0·8 mg/ml PHA, or abnormal (negative) when the maximal response is to a higher dose (4 mg/ml). The immunoglobulin classes G, A and M were measured using a standard radial immunodiffusion technique (Mancini *et al.*, 1965).

Patients failing to respond to treatment were excluded from further study when alternative treatment schedules were begun. Statistical analysis is by Student's *t* test or by Chi-square test as appropriate.

### RESULTS

Immunological assessment was carried out on all but the first 20 of the 92 patients described by Priestman *et al.* (1977). The remaining 72 were studied consecutively. Thirty-five patients received chemotherapy and 37 hormonal manipulation. Table I shows the pre-treatment assessment of the patients. There was no significant difference between the groups except that DNCB$^+$ and Mantoux$^+$ patients occurred more frequently in the chemotherapy group.

TABLE I.—*Comparison of endocrine and chemotherapeutic groups before treatment*

| | Chemotherapy (35) | Endocrine therapy (37) |
|---|---|---|
| Age (years) | 58±8 | 60±11 |
| DNCB$^{+*}$ | 20/28 (70%) | 15/31 (48%)† |
| Mantoux$^{+*}$ | 14/28 (50%) | 6/32 (19%)† |
| PHA$^{+*}$ | 14/30 (47%) | 15/31 (48%) |
| Total white-cell count (cells/mm$^3$ x 10$^{-3}$) | 6·49±1·29 | 7·06±2·52 |
| % lymphocytes | 21±12 | 17±8 |
| Lymphocyte count (cells/mm$^3$ x 10$^{-3}$) | 1·40±0·81 | 1·14±0·60 |
| IgG (mg/100 ml) | 1438±415 | 1491±375 |
| IgA (mg/100 ml) | 226±138 | 252±132 |
| IgM (mg/100 ml) | 134±83 | 134±90 |

* Number of positive/total observations.
† $P<0·05$ by Chi-square test.

TABLE II.—*DNCB reaction: No. positive/No. tested*

| | Chemotherapy | | Endocrine therapy | |
|---|---|---|---|---|
| | Response* | No response* | Response* | No response* |
| Pre-treatment | 13/14† | 7/14† | 4/7 | 11/24 |
| 6 weeks | 10/14 | 5/9 | 7/7 | 7/16 |
| 12 weeks | 9/13 | 2/5 | 5/7 | 4/9 |
| 26 weeks | 7/8 | 1/1 | 4/6 | 2/3 |

* In this and subsequent tables response/no response refers to objective response of tumour when assessed at 12 weeks.
† $P<0·05$ between response and no response to chemotherapy.

TABLE III.—*Mantoux reaction*

| | Chemotherapy | | Endocrine therapy | |
|---|---|---|---|---|
| | Response | No response | Response | No response |
| Pre-treatment | 7/13 | 7/14 | 2/7 | 4/24 |
| 6 weeks | 5/12 | 2/9 | 1/6 | 5/16 |
| 12 weeks | 4/13 | 1/5 | 2/7 | 1/9 |
| 26 weeks | 4/9 | 0/1 | 1/6 | 0/3 |

TABLE IV.—*PHA reaction of lymphocytes* in vitro*: No. with normal reaction/No. tested*

| | Chemotherapy | | Endocrine therapy | |
|---|---|---|---|---|
| | Response | No response | Response | No response |
| Pre-treatment | 7/17 | 7/13 | 4/5 | 11/26 |
| 6 weeks | 7/16 | 4/9 | 2/5 | 7/19 |
| 12 weeks | 5/15 | 5/9 | 3/5 | 5/7 |
| 26 weeks | 3/7 | 1/2 | 2/4 | 2/3 |

*Endocrine therapy*

Only 7 patients were classified as having responded to endocrine manipulation. None of the pre-treatment tests were able to predict a response to endocrine therapy, nor were there any statistically significant differences in immunological status (Tables II–X) during the period of observation. There was a trend for the IgM level to fall regardless of the clinical response, and this reached a statistically significant low level at 3 months when responders and non-responders are considered together. Patients responding to treatment restore their DNCB responses (Table II) to normal, and subsequently tend to revert to negative as their disease reappears.

*Chemotherapy*

Comparison of the 18 patients who responded to chemotherapy with those who did not (17 patients) shows that there were significantly ($P<0{\cdot}05$) more patients who were $DNCB^+$ (before treatment) amongst the responders (Table II). The pre-treatment values for the total white-cell count ($t=1{\cdot}69$; $P<0{\cdot}05$) shows that the responders had significantly lower levels, but no difference was found for the other variables (Tables III–X). There was a fall in the total white-cell count in both responders and non-responders after chemotherapy. The total white-cell count was significantly lower in the responders ($t=2{\cdot}76$; $P<0{\cdot}01$) at 6 weeks, and this difference was maintained throughout the study. Although there was a fall in the lymphocyte count this did not reach statistical significance until the last observation in the responding group (Table VII). The fall in the non-responders did not reach statistical significance. Again, a continuing fall in the levels of IgM was seen during the study, irrespective of the response to treatment, and this reached a statistically significant level ($P<0{\cdot}05$) after 12 weeks.

TABLE VI.—*% Lymphocytes: (mean ± s.d.)*

| | Chemotherapy | | Endocrine therapy | |
|---|---|---|---|---|
| | Response | No response | Response | No response |
| Pre-treatment | 22 ± 13 | 20 ± 13 | 16 ± 7 | 18 ± 10 |
| 12 weeks | 23 ± 11 | 26 ± 13 | 16 ± 9 | 18 ± 11 |
| 26 weeks | 18 ± 11 | 19 ± 3 | 13 ± 5 | 17 ± 15 |

The DNCB test was more frequently positive in those who responded to either treatment; 17/35 positive patients responded, while only 4/24 negative patients responded ($\chi^2=6{\cdot}32$; $P<0{\cdot}01$). None of the other tests in this profile had any predictive value in response.

### DISCUSSION

It is difficult to explain the differences in the immunological status of the two treatment groups before therapy. Since

TABLE V.—*Total white-cell counts* ($cells/mm^3 \times 10^{-3}$)*: mean ± s.d.*

| | Chemotherapy | | Endocrine therapy | |
|---|---|---|---|---|
| | Response | No response | Response | No response |
| Pre-treatment | 6·12 ± 1·16 | 6·92 ± 1·34 | 7·60 ± 1·68 | 6·94 ± 2·68 |
| 6 weeks | 4·45 ± 1·40* | 5·88 ± 0·47† | 7·84 ± 2·02 | 7·51 ± 2·90 |
| 12 weeks | 4·34 ± 1·27* | 5·91 ± 1·37† | 7·47 ± 2·36 | 6·35 ± 1·94 |
| 26 weeks | 4·90 ± 1·88* | 5·50 ± 0·89 | 7·77 ± 2·00 | 6·53 ± 1·67 |

* $P<0{\cdot}01$ Compared to pretreatment WBC in responders.
† $P<0{\cdot}05$ Compared to responders at the same time after treatment started.

TABLE VII.—*Absolute lymphocyte count* (*cells/mm*$^3$ × $10^{-3}$): *mean* ± *s.d.*

| | Chemotherapy | | Endocrine therapy | |
|---|---|---|---|---|
| | Response | No response | Response | No response |
| Pre-treatment | 1·37±0·78 | 1·43±0·87 | 1·21±0·67 | 1·13±0·60 |
| 6 weeks | 0·96±0·45 | 1·39±0·63 | 1·16±0·48 | 1·23±0·64 |
| 12 weeks | 0·95±0·58 | 1·16±0·95 | 1·28±1·04 | 1·03±0·42 |
| 26 weeks | 0·79±0·51* | 1·15±0·07 | 1·03±0·47 | 1·15±0·78 |

* $P < 0·05$ with respect to pre-treatment values.

TABLE VIII.—*Immunoglobulin—IgG: mean* ± *s.d.* (*mg*/100 *ml*)

| | Chemotherapy | | Endocrine therapy | |
|---|---|---|---|---|
| | Response | No response | Response | No response |
| Pre-treatment | 1464±471 | 1405±343 | 1275±373 | 1539±365 |
| 6 weeks | 1332±371 | 1435±277 | 1319±194 | 1519±382 |
| 12 weeks | 1361±388 | 1195±262 | 1407±291 | 1392±320 |
| 26 weeks | 1311±453 | 1081±339 | 1307±309 | 1384±565 |

TABLE IX.—*Immunoglobulin—IgA* (*mg*/100 *ml*): *mean* ± *s.d.*

| | Chemotherapy | | Endocrine therapy | |
|---|---|---|---|---|
| | Response | No response | Response | No response |
| Pre-treatment | 191±82 | 272±181 | 270±179 | 248±128 |
| 6 weeks | 182±62 | 196±89 | 243±112 | 217±98 |
| 12 weeks | 187±65 | 189±100 | 270±141 | 212±84 |
| 26 weeks | 178±79 | 253±135 | 267±153 | 228±155 |

TABLE X.—*Immunoglobulin—IgM* (*mg*/100 *ml*): *mean* ± *s.d.*

| | Chemotherapy | | Endocrine therapy | |
|---|---|---|---|---|
| | Response | No response | Response | No response |
| Pre-treatment | 125±56 | 146±109 | 117±111 | 136±88 |
| 6 weeks | 97±52 | 103±50 | 91±54 | 137±76 |
| 12 weeks | 85±37* | 69±47* | 77±41 | 96±49 |
| 26 weeks | 73±37* | 71±37 | 87±56 | 92±37 |

* $P < 0·05$ with respect to pre-treatment values.

the treatments were randomly allocated and the patients studied were consecutively entered into the trial, it is possibly a chance finding related to the small numbers studied.

With the sole exception of the DNCB response, the tests used in the immunological profile failed to identify the patients likely to respond to therapy. This finding may be equated with the observation that only the DNCB response correlated with disease stage in breast cancer (Bolton *et al.*, 1976). In a similar way we found that in patients receiving chemotherapy a significant lowering of the total white-cell and lymphocyte counts is associated with a clinical response (O'Bryan *et al.*, 1977). Whether this is a reflection of the closeness of toxic and therapeutic doses, or whether the lowering of the white-cell count is an epiphenomenon secondary to a reduction in tumour bulk remains unclear. Mott (1973) advanced the hypothesis that immunodepression occurring during chemotherapy might be beneficial, perhaps by exerting a specific effect on suppressor cells. Our findings that the peripheral white-cell count and lymphocyte count fall significantly would be consistent with this hypothesis.

Whilst the effect of chemotherapy on the immune system, especially in respect of transplantation, has been extensively studied, the effects of hormonal manipulation on lymphocyte numbers and function have been relatively ignored, although the effect on the monocyte/macrophage system has been more extensively studied (Baum, 1975). Yonemoto *et al.* (1977) have demonstrated that a good clinical response to adrenalectomy was associated with a rise in T-cell count and a decrease in blocking factor, but were unable to elucidate cause and effect. Our studies have shown little change in immune status of patients who respond, in spite of their improved clinical state. Franks *et al.* (1978) have recently reported that patients with a low level of circulating lymphocytes are less likely to respond to hormone therapy. However, he did not demonstrate any significant change in levels with treatment.

The progressive fall in IgM in both treatment groups during the study remains an enigma, since it has not been found to relate to disease stage (Bolton *et al.*, 1976) nor does it appear to be related to treatment or response in this study. It does not seem to be a laboratory-related phenomenon, since the results from the laboratory controls did not change during the period of the study.

We find that this chemotherapeutic regime is lacking in severe short-term immunosuppressive effects, and indeed clinical improvement is associated with increased DNCB reactivity in most patients in remission at 6 months. The long-term effects of the myelosuppression remain unknown, but there seems to be a trend for the white-cell count to increase with time.

We gratefully acknowledge the help of Dr R. H. Whitehead and Dr J. W. Keyser with the laboratory studies.

### REFERENCES

ARSENEAU, J. C., SPONZO, R. W., LEVIN, D. L. & 6 others (1972) Non lymphomatous malignant tumours complicating Hodgkin's disease. *N. Engl. J. Med.*, **287**, 119.

BAUM, M. (1975) In *Host Defences in Breast Cancer*. Ed. B. A. Stoll. Chicago: Year Book Medical Publishers. p. 130.

BOLTON, P. M., TEASDALE, C., MANDER, A. M. & 5 others (1976) Immune competence in breast cancer. Relationship of pretreatment immunologic tests to diagnosis and tumour stage. *Cancer Immunol. Immunother.*, **1**, 251.

EDELSTYN, G. A. & MACRAE, K. D. (1973) Cyclical combination chemotherapy in advanced breast cancer. *Br. J. Cancer*, **28**, 459.

FRANKS, C. R. & WILLIAMS, Y. (1978) The effect of sex hormones on peripheral immunity in patients with advanced breast cancer. *Clin. Oncol.*, **4**, 19.

HARRIS, C. C. (1975) Immunosuppressive anticancer drugs in man, their autologic potential. *Radiology*, **114**, 163.

MANCINI, G., CARBONERA, A. O. & HEREMANS, J. F. (1965) Immunochemical quantitation of antigens by single radial immunodiffusion. *Immunochemistry* **2**, 235.

MOTT, M. G. (1973) Chemotherapeutic suppression of immune enhancement—a primary determinant of successful cancer therapy. *Lancet*, i, 1092.

O'BRYAN, R. M., BAKER, L. H., GOTTLIEB, J. E. & 6 others (1977) Dose response evaluation of adriamycin in human neoplasia. *Cancer*, **39**, 1940.

PRIESTMAN, T., BAUM, M., JONES, V. & FORBES, J. F. (1977) Comparative trial of endocrine *versus* cytotoxic treatment in advanced breast cancer. *Br. Med. J.*, i, 1248.

SUTHERLAND, R. M., MCCREDIE, J. A. & INCH, W. R. (1975) Effect of splenectomy and radiotherapy on lymphocytes in Hodgkin's disease. *Clin. Oncol.*, **1**, 2751.

WHITEHEAD, R. H., BOLTON, P. M., NEWCOMBE, R. G., JAMES, S. L. & HUGHES, L. E. (1975) Lymphocyte response to PHA in breast cancer: correlation of predicted prognosis to response to different PHA concentrations. *Clin. Oncol.*, **1**, 191.

WILSON, E. R., HAGER, E. B., HAMPERS, G. L., CARSON, J. M., MERRILL, J. P. & MURRAY, J. E. (1968) Immunologic rejection of human cancer transplanted with renal allograft. *N. Engl. J. Med.*, **278**, 479.

YONEMOTO, R. H., SCHICK, P., ALBANO, W., FUJISAWA, T. & WALDEMAN, S. H. (1977) Immune responses in the treatment of advanced carcinoma of the breast—effects of adrenalectomy. *Arch. Surg.*, **112**, 991.